# Identification of Plasma Lipid Alterations Associated with Melanoma Metastasis

**DOI:** 10.3390/ijms25084251

**Published:** 2024-04-11

**Authors:** István Szász, Viktória Koroknai, Tünde Várvölgyi, László Pál, Sándor Szűcs, Péter Pikó, Gabriella Emri, Eszter Janka, Imre Lőrinc Szabó, Róza Ádány, Margit Balázs

**Affiliations:** 1 HUN-REN-UD Public Health Research Group, Department of Public Health and Epidemiology, Faculty of Medicine, University of Debrecen, 4028 Debrecen, Hungary; szasz.istvan@med.unideb.hu (I.S.); roza.adany@med.unideb.hu (R.Á.); 2 Department of Public Health and Epidemiology, Faculty of Medicine, University of Debrecen, 4028 Debrecen, Hungary; koroknai.viktoria@med.unideb.hu (V.K.); pal.laszlo@med.unideb.hu (L.P.); szucs.sandor@med.unideb.hu (S.S.); piko.peter@med.unideb.hu (P.P.); 3 Department of Dermatology, Faculty of Medicine, University of Debrecen, 4032 Debrecen, Hungary; varvolgyi.tunde@med.unideb.hu (T.V.); gemri@med.unideb.hu (G.E.); janka.eszter@med.unideb.hu (E.J.); szabo.imre@med.unideb.hu (I.L.S.)

**Keywords:** melanoma, metastasis, plasma lipid profile, biomarkers, lipidyzer platform

## Abstract

The aim of this study was to apply a state-of-the-art quantitative lipidomic profiling platform to uncover lipid alterations predictive of melanoma progression. Our study included 151 melanoma patients; of these, 83 were without metastasis and 68 with metastases. Plasma samples were analyzed using a targeted Lipidyzer™ platform, covering 13 lipid classes and over 1100 lipid species. Following quality control filters, 802 lipid species were included in the subsequent analyses. Total plasma lipid contents were significantly reduced in patients with metastasis. Specifically, levels of two out of the thirteen lipid classes (free fatty acids (FFAs) and lactosylceramides (LCERs)) were significantly decreased in patients with metastasis. Three lipids (CE(12:0), FFA(24:1), and TAG47:2-FA16:1) were identified as more effective predictors of melanoma metastasis than the well-known markers LDH and S100B. Furthermore, the predictive value substantially improved upon combining the lipid markers. We observed an increase in the cumulative levels of five lysophosphatidylcholines (LPC(16:0); LPC(18:0); LPC(18:1); LPC(18:2); LPC(20:4)), each individually associated with an elevated risk of lymph node metastasis but not cutaneous or distant metastasis. Additionally, seventeen lipid molecules were linked to patient survival, four of which (CE(12:0), CE(14:0), CE(15:0), SM(14:0)) overlapped with the lipid panel predicting metastasis. This study represents the first comprehensive investigation of the plasma lipidome of melanoma patients to date. Our findings suggest that plasma lipid profiles may serve as important biomarkers for predicting clinical outcomes of melanoma patients, including the presence of metastasis, and may also serve as indicators of patient survival.

## 1. Introduction

It is widely accepted that melanoma has the highest mutational burden of all cancer types [1], leading to distinct metabolic differences between melanoma and normal cells. These mutations result in alterations in metabolic pathways that enable tumor cells to survive in a constantly changing environment [2]. The Warburg effect is a metabolic abnormality that has been recognized as one of the oldest features of cancer cells. It is a peculiar process of energy production in which cancer cells obtain energy in an unusual, inefficient way. Rather than breaking down glucose molecules by oxidative phosphorylation using oxygen they ferment without oxygen, similar to yeast cells. This process not only produces energy but also provides a sufficient carbon source to synthesize the building blocks of proteins, nucleotides, and lipids [3,4].

Research on altered lipid metabolism in cancer cells dates back to the 1960s when it was observed that tumor cells actively synthesize and uptake lipids [5,6]. Subsequently, it was discovered that a highly expressed gene in breast cancer encodes fatty acid synthase (FASN), a key enzyme in lipid metabolism [7]. Numerous studies have since confirmed that alterations in lipid metabolism are important processes in tumor progression, involving not only tumor cells but also other cell types in the tumor microenvironment, such as stromal and endothelial cells [8,9,10,11,12].

One of the major differences between normal and tumor cells is that tumor cells are highly dependent on a constant supply of fatty acids (FAs) and cholesterol, as they need to synthesize large quantities of membranes for continuous growth, proliferation, and metastasis [13]. In addition, while tumor cells retain their ability to acquire lipids from the circulation and adjacent adipose tissue, they also synthesize the majority of their lipids de novo, as evidenced by the overexpression of necessary enzymes [8]. Alterations in lipid metabolism and changes in lipid composition can serve as valuable biomarkers in various cancers, including melanoma [14]. Differences in lipid profiles and variations in the expression of lipid metabolism-related enzymes between cancerous and noncancerous tissues have been identified as possible cancer biomarkers [15]. In addition, recent research has identified specific lipid patterns associated with disease stage, prognosis, or response to treatment [16,17]. Dei Cas et al., described lipid species alterations characterizing the signature of melanoma kinase inhibitor resistance in plasma melanoma patients, indicating the significance of dysregulated lipid metabolism in melanoma patients. Technological advancements now enable simultaneous quantitative and qualitative characterization of thousands of lipids, greatly facilitating the study of the relationship between altered lipid metabolism and pathological processes [17].

In this study, we used a state-of-the-art quantitative lipidomics profiling platform, Lipidyzer™, which covers over 1100 lipid species across 13 lipid classes. Our objective was to conduct the most comprehensive investigation to date of the plasma lipidome to uncover lipid alterations predictive of melanoma progression. Our study involved 151 melanoma patients, comprising 83 without metastasis and 68 with metastases. Our findings suggest that plasma lipid alterations may serve as significant biomarkers for predicting clinical outcomes in melanoma patients, including metastasis, and as indicators of patient survival.

## 2. Results

### 2.1. Distribution of Lipid Classes between Melanoma Patients without and with Metastasis

**Figure 1:**
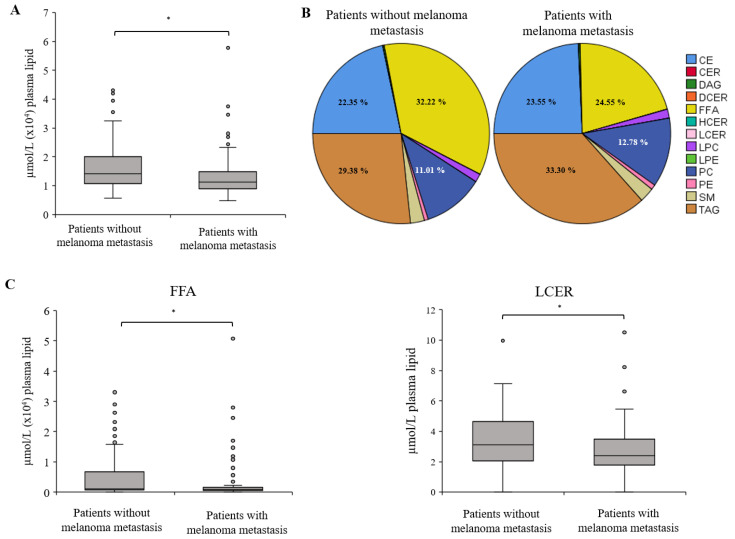
Comparison of lipid species distribution in plasma between metastasis-free and patients with melanoma metastasis. (**A**) A quantitative comparison of total lipid content in melanoma patient plasma revealed 802 lipids across 13 lipid classes, identified in both metastasis-free and metastatic melanoma patients (*p* < 0.002). (**B**) A pie chart illustrates the distribution of lipid classes between metastasis-free patients and those with melanoma metastasis. (**C**) Significantly altered lipid classes (FFA and LCER) were observed in patients with metastatic disease compared to the metastasis-free group (*p* < 0.03). * significant *p*-value (*p* < 0.05). CE: cholesteryl ester; CER: ceramide; DAG: diacylglycerol; DCER: dihydroceramide; FFA: free fatty acid; HCER: hexosylceramide; LCER: lactosylceramide; LPC: lysophosphatidylcholine; LPE: lysophosphatidylethanolamine; PC: phosphatidylcholine; PE: phosphatidylethanolamine; SM: sphingomyelin; TAG: triacylglycerol.

We analyzed peripheral blood plasma samples from 151 melanoma patients using the Sciex Lipidyzer™ platform. Of these, 83 patients had their primary melanoma removed at least one month prior to blood sampling and were considered tumor-free. All patients underwent a negative CT scan at least one month prior to blood sampling, confirming the absence of metastasis. Sixty-eight patients were diagnosed with melanoma metastasis. Details of the clinical parameters of the patients and the tumor samples are summarized in the Section 4 (Table 1). After the implementation of quality control filters, a total of 802 lipid species from 13 lipid classes were detected in the plasma samples and included in the subsequent analyses. Our initial observation revealed that the total concentration of the 802 lipid species in patients’ samples with metastases was significantly lower compared to those without metastasis (*p* ≤ 0.05) (Figure 1A).

### 2.2. Association between Lipid Species and the Presence of Metastasis in Melanoma Patients

**Figure 2:**
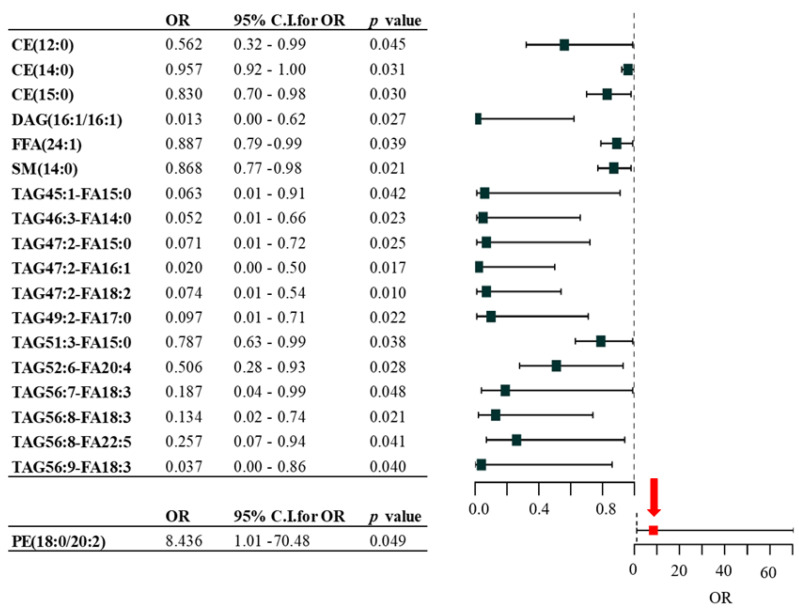
Association of lipid species with the presence of melanoma metastasis. Binary logistic regression analysis was used. Odds ratios and confidence intervals are visualized on forest plot. Black square labeled lipids show negative; red square labeled lipids show positive association with metastasis (indicated by red arrow). OR: odds ratio; C.I.: confidence interval adjusted by sex, age, and therapy; CE: cholesteryl ester; DAG: diacylglycerol; FFA: free fatty acid; PE: phosphatidylethanolamine; SM: sphingomyelin; TAG: triacylglycerol.

To investigate the association between lipid species and the presence/absence of metastatic tumors, a logistic regression model adjusted for gender, age, and type of therapy was performed. This analysis identified 19 lipid species with significant prognostic value (Figure 2). We found that patients plasma with a lower level of lipid species (18 different lipids) and a higher level of the PE(18:0/20:2) lipid molecule were more likely to have melanoma metastasis (ORs, 95% C.I.s, and *p* values are displayed in Figure 2).

**Figure 3:**
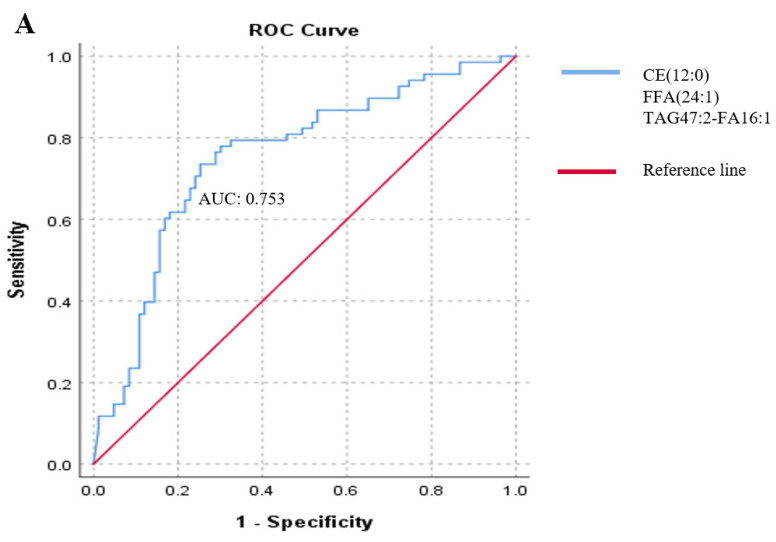

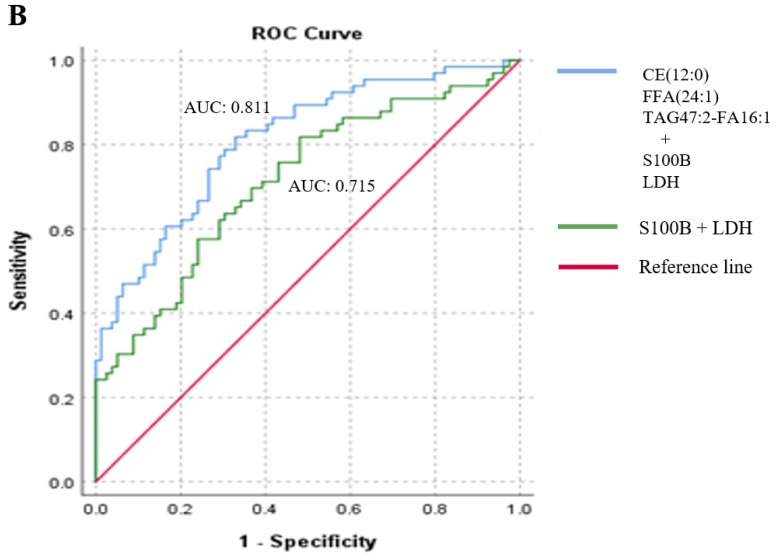
Association of three lipid species and melanoma markers with the presence of melanoma metastases. During this analysis, receiver operating characteristic (ROC) classifier curves were used. (**A**) ROC curve analysis of three lipids (CE(12:0), FFA(24:1), and TAD47:2-FA16:1) associated with metastasis resulting from stepwise logistic regression (blue line), and the red line indicates the reference line. (**B**) ROC curve analysis of the three lipids combined with S100B and LDH melanoma markers associated with metastasis. The blue line represents three metastasis-associated lipids combined with S100B and LDH melanoma markers, the green line represents S100B and LDH melanoma markers, and the red line indicates the reference line. CE: cholesteryl ester; FFA: free fatty acid; TAG: triacylglycerol.

We applied forward stepwise logistic regression to identify the strongest associations among the 19 significant lipids with the presence of metastasis. The analysis revealed a panel of three lipids (CE(12:0), FFA(24:1), and TAG47:2-FA16:1) whose association was independent of patient age, sex, and therapy. To determine the likelihood of this lipid panel predicting metastasis, we generated a receiver operating characteristic (ROC) curve. The area under the curve (AUC) value of the ROC analysis was 0.753 (Figure 3A).

We also examined the potential role of this lipid panel in predicting metastasis by combining the levels of these three lipids with the plasma levels of serum lactate dehydrogenase (LDH) (as recommended by the American Joint Committee on Cancer (AJCC) revised melanoma staging guidelines) and the calcium-binding acidic cytoplasmic protein S100B, which is a well-known melanoma marker. The combination of these five markers resulted in an improved AUC score of 0.811 in the analysis, compared to an AUC of 0.715 for the conventional S100B and LDH model (Figure 3B).

### 2.3. Lipid Species Associated with the Lymphatic and Hematogenous Pathways of Melanoma Metastasis

**Figure 4:**
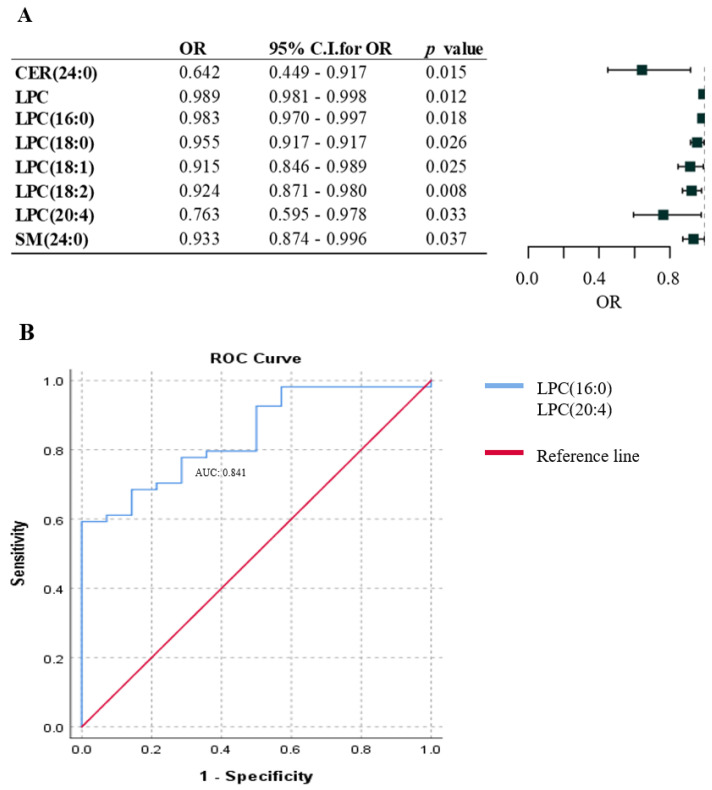
Association of lipid species with lymphatic and hematogenous pathways of melanoma metastasis. (**A**) Binary logistic regression analysis of lipid species in association with the lymphatic and hematogenous pathways of melanoma metastasis. Odds ratios and confidence intervals are visualized on forest plot. Black squared lipids show a negative association with the hematogenous pathway of metastasis. (**B**) ROC curve analysis of the seven lipids by stepwise regression resulted in two lipids (LPC(16:0) and LPC(20:4)) that were strongly associated with the hematogenous pathway of melanoma metastasis. OR: odds ratio; C.I.: confidence interval (adjusted by sex, age, and therapy). Blue line represents the two lysophosphatidylcholines (LPCs); red line indicates the reference line.

To investigate the association between lipid species and different pathways of melanoma metastasis, we divided plasma samples derived from patients with metastases into two groups. The first group included patients with lymph node metastasis (n = 19), while the second group involved patients with hematogenous metastases (n = 49) classified according to these two pathways. Figure 4 summarizes the lipid species associated with the two different pathways of metastasis in melanoma patients. The total amount of LPC and seven different lipids (including five LPCs: (LPC(16:0), LPC(18:0), LPC(18:1), LPC(18:2), and LPC(20:4)) showed a negative association with the hematogenous pathway, indicating that reduced levels of these lipids were associated with distant metastases (Figure 4A).

To provide a quantitative tool for predicting the individual probability of lymphatic metastasis risk, a different approach was applied using the five LPC lipid species ((LPC(16:0), LPC(18:0), LPC(18:1), LPC(18:2), and LPC(20:4)) that were associated with the metastatic pathway (Figure 4A). The combined values of the five lipids were investigated using ROC analysis, which revealed a plasma concentration cutoff point of 202.46 µmol/L, yielding a sensitivity of 0.895 and a specificity of 0.510. Samples were stratified based on the cutoff level, and logistic regression analysis indicated those patients with a summed plasma concentration of the five lipids > 202.46 µmol/L had a 90.7% higher probability of lymphatic metastasis compared to hematogenous metastasis (*p* = 0.004; odds ratio: 0.093; 95% confidence interval: 0.018–0.477).

Stepwise regression analysis resulted in a panel of two lipids, LPC(16:0) and LPC(20:4), which exhibited remarkably predictive capability for the type of metastasis pathway, achieving an AUC score of 0.841 as determined by ROC analysis. The predictive value of these two lipids did not increase when they were combined with additional lipids from the LPC class (Figure 4B).

**Figure 5:**
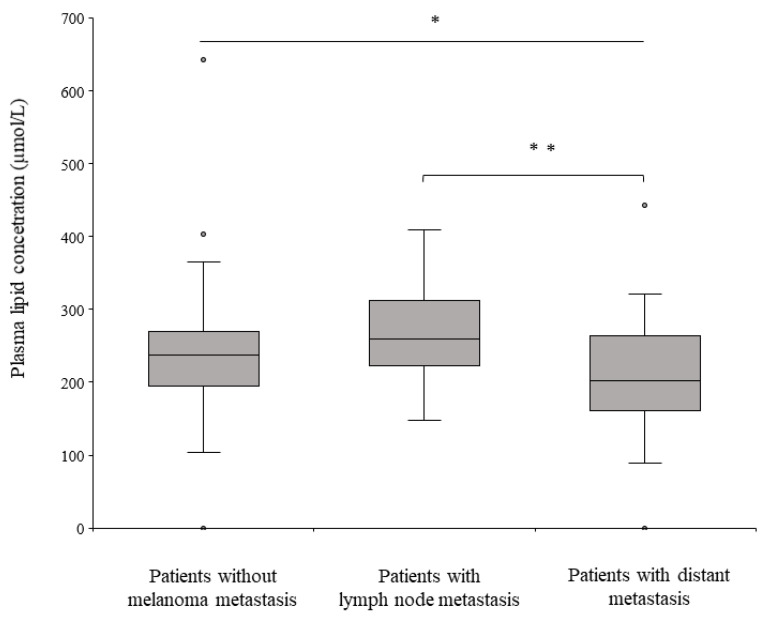
Plasma lipid concentrations in three different groups of melanoma patients. Quantitative difference in five LPCs (LPC(16:0), LPC(18:0), LPC(18:1), LPC(18:2), and LPC(20:4)) associated with different types of melanoma metastasis. The results were obtained by the Kruskal–Wallis test. The results of Dunn’s post hoc test are presented above the bar, indicating significance levels (* *p* < 0.05; ** *p* < 0.01).

Additionally, we investigated the variations in the levels of these five LPCs among patients without metastasis, those with lymph node metastases, and those with distant metastases. The levels of these lipids were elevated in patients with lymph node metastases and decreased in patients with distant metastases. The results are summarized in Figure 5.

### 2.4. Lipid Species Associated with Melanoma Patient’s Survival

**Figure 6:**
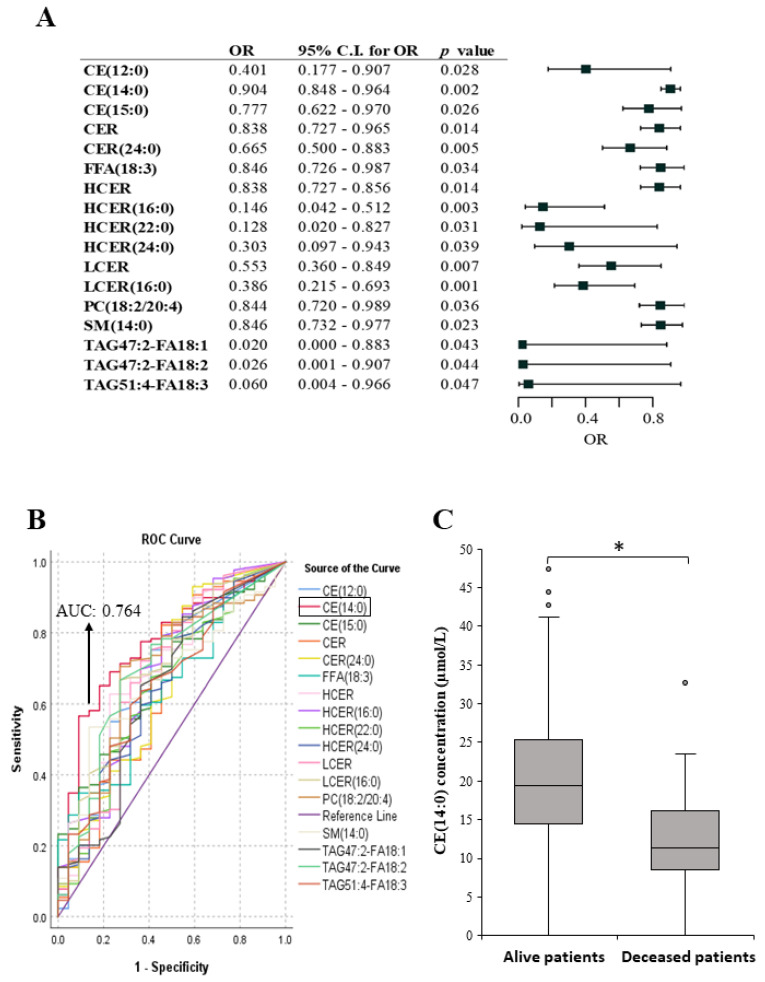
Binary logistic regression analysis examining the relationship between lipid species and the mortality of melanoma patients. (**A**) Odds ratios and confidence intervals are visualized by forest plot. Black squares indicate a negative association with patient mortality, while (**B**) shows ROC curve analysis of the lipid panel associated with patient mortality. The highest AUC (0.764) was detected for the CE(14:0) lipid species. (**C**) Quantitative changes in the CE(14:0) lipid level of deceased (n = 22) and living (n = 129) patients. Mann–Whitney Wilcoxon test; * *p* = 0.02. OR: odds ratio; C.I.: confidence interval; CE: cholesteryl ester; CER: ceramide; FFA: free fatty acid; HCER: hexosylceramide; LCER: lactosylceramide; PC: phosphatidylcholine; SM: sphingomyelin; TAG: triacylglycerol.

Despite the small number of deceased patients in our study group (22 patients died within 3 years out of the 151 patients at the time of analysis), we also examined the lipid fingerprints of both the alive and deceased groups. A decreased level of seventeen lipid molecules was associated with patients who died within 3 years after the diagnosis of melanoma metastases (Figure 6A). Stepwise regression analysis revealed that one lipid (CE(14:0)) exhibited the highest AUC among all the metastasis-associated lipids (Figure 6B, red line), even when used in combination with other lipids. The average CE(14:0) plasma lipid levels in deceased and surviving patients are depicted in Figure 6C. Based on the concentration of the CE(14:0) lipid, a cutoff value of 18.03 µmol/L was determined in the plasma of melanoma patients, with those above this threshold being eleven times more likely to survive (*p* = 0.002; OR: 11.487; CI 2.501–52.761).

## 3. Discussion

Dysregulated lipid metabolism is frequently observed in various types of cancer [18]. This phenomenon can be exploited in two ways. One approach is to target the resistant tumors that most often interfere with the efficacy of therapy through altered lipid pathways. This applies to both targeted therapy and immunotherapy [19,20,21]. The other approach is to consider them as potential biomarkers to improve diagnosis, monitor disease progression, and predict outcomes [22,23]. In our current study, we aimed to identify lipid markers associated with melanoma metastasis, the type of metastatic pathway (lymphogenic or hematogenous), and patient mortality.

When describing changes in plasma lipids in cancer patients, it is common to focus on alterations in cholesterol. Notably, cancer patients often exhibit reduced levels of HDL and an increased risk of metastasis associated with elevated LDL levels [24,25,26,27]. Our data reveal significant reductions in total plasma lipid levels in patients with melanoma metastases, along with alterations in the percentage distributions within the 13 lipid classes. Among these lipid classes, we noted significant decreases in free fatty acid and long-chain ceramide levels in melanoma patients with metastasis. The decrease in FFA levels may seem surprising, as several studies have reported elevated FFA levels in various cancers, including prostate, lung, gastric, thyroid, colorectal, and ovarian cancers, as well as B-cell lymphoma [28,29]. However, breast cancer is associated with decreased levels of FFAs [30], and based on our data, melanoma appears to fall into this category. The levels of lactosylceramides were also significantly reduced in the plasma of metastatic patients. This class of sphingolipids is known to be associated with increased cell survival, proliferation, adhesion, and invasion, thereby promoting tumor progression [31,32].

Functionally, lipids are involved in several stages of the metastatic cascade. In different types of cancers, the overexpression of genes related to fatty acid uptake, lipid accumulation, and other fatty acid metabolism processes has been linked to increased invasiveness, migratory properties, and the ability of tumor cells to proliferate in distant organs [33,34,35]. These alterations are associated with metastatic progression and poor prognosis in various cancer patients [34]. In many cases, plasma lipid levels were inversely related to the presence of metastasis and a worse prognosis, with only a few exceptions. The most plausible explanation for this phenomenon is that these lipids accumulate in tumor cells and support their survival and proliferation, thereby limiting their release into the bloodstream. Excess cellular cholesterol levels are regulated by the cholesterol-synthesizing enzyme 3-hydroxy-3-methyl-glutaryl-CoA reductase (HMGCR). They are converted to cholesteryl esters by the enzyme acetyl-coenzyme A cholesterol acetyltransferase 1 (ACAT1) and removed from the intracellular spaces by transporters such as ATP-binding cassette A-1 (ABCA1) [36,37,38].

In a study by Hartmann et al., enzymes responsible for intracellular cholesterol ester accumulation (HMGCR and ACAT1) were overexpressed, and the expression of the transporter responsible for their release (ABCA1) was downregulated in lung tumors [39]. This supports our findings that reduced plasma levels of various cholesterol esters, such as CE(12:0), CE(14:0), and CE(15:0), were associated with a higher probability of metastasis and, not surprisingly, patient mortality. This phenomenon is in line with our observations on other lipids. Notably, we also observed a significant decrease in the levels of several TAG lipid species in plasma samples from metastatic melanoma patients, similar to the reduced serum TAG levels in hepatocellular carcinoma [40,41]. This reduction may be due to pro-inflammatory cytokines produced by the tumor, such as IL-1, which delay intestinal absorption and reduce tissue lipid uptake. Interleukin-2, however, can induce hypocholesterolemia by inhibiting the activity of lecithin-cholesteryl acyltransferase (LCAT) [42,43].

As tumors can metastasize by intravasation into venous capillaries (hematogenous pathway) or the lymphatic system (lymphatic pathway), the identification and validation of markers that influence the intravasation process have important clinical implications for prognosis and treatment. Lysophosphatidylcholine (LPC) is derived from PCs by phospholipase A2. It is degraded to glycerophosphocholine and free FA (FFA) and catalyzed by extracellular lysophospholipases A1 and A2 [44]. LPC can modulate ion concentrations, including Ca2+, Na+, and K+, by binding to G protein-coupled and Toll-like receptors. Importantly, LPC can induce lymphocyte and macrophage migration, increase the production of proinflammatory cytokines, induce oxidative stress, and promote apoptosis, inflammation, and disease development [45]. It is known that plasma phospholipid and LPC levels decrease in several cancers, including prostate cancer, acute leukemia, and lung cancer, and that this decrease correlates with tumor progression, making it a promising biomarker [24,46,47,48,49,50]. In our study, we observed that the total number of LPCs was higher in patients with lymphatic metastases compared to patients without metastasis but the lowest level was detected in plasma of patients who had distant metastasis. These prominent LPC species, such as palmitoyl, oleoyl, linoleoyl, and arachidonoyl-lysophosphatidylcholine (LPC 16:0, 18:1, 18:2, and 20:4), serve as predictive markers for lymph node metastasis rather than hematogenous metastasis. These LPCs are among the most abundant in human plasma [51] and all are capable of inducing the expression of COX-2 [52], which is known to be associated with lymph node metastasis in several cancers [53,54]. This involvement of lymph nodes can be explained by the fact that while COX-1 maintains a constant expression as a housekeeping gene, COX-2 is rapidly inducible, tightly regulated, and significantly upregulated during inflammation [55]. Previous studies have highlighted the reduction in invasiveness associated with COX2 inhibition, making it a promising target [56,57,58]. Our research suggests that the development of lymph node metastasis begins at the level of fatty acids and is significantly related to the LPCs we have described. Consequently, the invasiveness of cells can also be modulated by inhibiting LPCs, most likely at a slightly earlier stage.

In our cohort, several lipid classes were found to be different between deceased and surviving patients. Reduced CE(14:0) levels were associated with a significantly higher risk of death. This finding is similar to that of other cholesteryl esters, including CE(12:0) and CE(15:0), which were also found when comparing metastasis-free and metastatic patients. In the blood plasma, cholesterol exists in two forms, free cholesterol (Chol) and cholesteryl esters (CEs). The esterification of Chol to CE occurs in the endoplasmic reticulum of both intestine and liver cells and is catalyzed by ACAT [59]. These changes in CE levels may be attributed to alterations in the expression of genes involved in the metabolism of CE. For example, in colorectal cancer (CRC), Liu et al. reported significantly higher levels of lysosomal acid lipase (LAL), which is responsible for the hydrolysis of CE in tumor patients. Conversely, decreased levels of ACAT1, which is responsible for CE synthesis, were negatively correlated with CRC progression. Lower ACAT1 immuno-histochemical (IHC) scores were associated with more advanced clinical stages of CRC [60]. Notably, nearly 80% of advanced cancer patients suffer from a severe wasting syndrome, known as cancer cachexia. Cachexia is characterized by significant weight loss due to loss of skeletal muscle and adipose tissue [61], which is also associated with decreased cholesterol levels [62]. Last but not least, we identified four lipids (CE(12:0), CE(14:0), CE(15:0), SM(14:0)) that overlapped between the lipid species of patients with and without metastatic tumors and between deceased and living patients. These overlaps in the lipid patterns confirm the reliability of our results.

In summary, we have, for the first time, identified numerous lipidomic abnormalities in the plasma of melanoma patients that have diagnostic and predictive value. We encourage further research in this field. It is crucial to emphasize that to comprehensively understand the lipidomic changes associated with tumorigenesis, simultaneous study of lipid patterns in normal and tumor tissue as well as plasma/serum is essential. This is due to the potential correlation between a decrease in the amount of a particular lipid in one location and an increase in the same lipid in another location (e.g., tumor–normal tissue) and vice versa [63].

## 4. Materials and Methods

### 4.1. Characteristics of Patients and Melanoma Samples Included in the Study

All tumor samples were handled according to the rules and regulations of the University of Debrecen, Hungary, with the approval of the Ethics Committee of the Hungarian Scientific Council for Health (TUKEB 17876–2018/EKU and BMEÜ/715-1 /2022/EKU). One hundred fifty-one melanoma patients were included in this study. Characteristics of the tumor samples are summarized in Table 1. Eighty-three patients were considered virtually metastasis-free, having had a negative CT scan at least one month prior to blood sampling, indicating the absence of metastasis. Additionally, 68 patients already had metastatic melanoma at the time of blood sampling. The patients involved 84 males (56%) and 67 (44%) females, with a median age of 61.91 years (range 29–84 years). Out of the 84 male patients (55.6%), 45 (53.5%) were diagnosed with melanoma metastases (11 had lymph node metastasis and 34 had distant metastases) and 34.3% of the female patients (n = 23) developed metastases (8 patients had lymph node metastasis and 15 had distant metastases) (Table 1). The distant metastases mainly affected the lungs, brain, liver, or kidneys. Based on the tumor samples, we have categorized two groups according to the pathway of metastasis: lymphatic and hematogenous. Lymph node metastases were present in 19; distant and/or cutan metastases were detected in 49 patients at the time of blood collection. The subtypes of melanoma from patients considered tumor-free at the time of blood collection were cutaneous (98.7%), and 2 primary tumors were choroidal melanomas. It is important to note that patients with advanced metastases almost always exhibit lymph node involvement, making it challenging to entirely separate the two groups (Table 1).

**Table 1:** Characteristics of melanoma patients and tumor samples.

			Type of Therapy at the Time of Blood Sampling		
	Number of Patients (%)	Metastases Peresent (%)	None (%)	Immuno-Therapy ^a^ (%)	Targeted Therapy ^b^ (%)
All patients	151 (100)	68 (45.0)	64 (43.4)	64 (43.4)	23 (15.2)
Gender					
Female	67 (44.4)	23 (34.3)	31 (46.2)	27 (40.3)	9 (13.4)
Male	84 (55.6)	45 (53.6)	33 (33.3)	37 (44.0)	14 (16.7)
Age (years) (average age: 61.91)					
20–50	28 (18.5)	8 (28.6)	14 (50.0)	9 (32.1)	5 (17.9)
≥50	123 (81.5)	60 (48.8)	50 (40.7)	55(44.7)	18 (14.6)
Metastasis					
Absent	83 (55.0)	83 (55.0)	53 (63.9)	22 (26.5)	8 (9.6)
Present	68 (45.0)	68 (45.0)	11 (16.2)	42 (61.8)	15 (22.1)
Pathway of metastasis					
Lymphatic (only lymph node)	19 (12.6)				
Hematogenous (distant and/or cutan)	49 (32.5)				
Patient’s survival (with a 3-year follow-up period)					
Alive	129 (85.4)				
Deceased	22 (14.6)				

^a^ Immunotherapies: OPDIVO^®^ (nivolumab); OPDIVO^®^ (nivolumab) + YERVOY^®^ (ipilimumab); Keytruda (pembrolizumab). ^b^ Targeted therapies: TAFINLAR^®^ (dabrafenib)+MEKINIST^®^ (trametinib); Zelboraf (vemurafenib) + COTELLIC^®^ (cobimetinib).

### 4.2. Blood Samples

Blood samples were collected at the Department of Dermatology, Faculty of Medicine, University of Debrecen, Hungary. Blood sampling was performed at least one month after surgical removal of the primary tumor or while patients had metastatic melanoma. All blood specimens were processed within one hour postdraw. The blood was collected into BD Vacutainer^®^ Venous Blood Collection Tubes (cat. no. 367525) containing EDTA. It was then transferred to 15 mL Falcon tubes and centrifuged at 4 °C and 3000 rpm for 10 min. The supernatant was then carefully placed into 2 mL Eppendorf tubes and centrifuged at 4 °C and 16,000 rpm for 10 min. Specimens were shipped to the Institute of Public Health and Epidemiology on dry ice and were stored at −80 °C until use.

### 4.3. Standards and Extraction of Lipids

Methanol, 2-propanol, dichloromethane, water, and ammonium acetate were purchased from VWR International, LLC (Radnor, Radnor, PA, USA). All of these products were of HPLC grade. Internal standard (ISTD) kits (containing ISTDs for 13 lipid classes), pike standards with quality control plasma kits, SelexION tuning kits, and system suitability test kits for quantitative lipidomic analysis of human samples were purchased from AB Sciex Germany GmbH (Darmstadt, Germany). The composition of ISTD standard mixtures containing isotope-labeled lipid molecules was previously described in detail [64,65]. Lipids were extracted from the plasma samples using a modified Bligh-Dyer method [66].

### 4.4. Lipidomic Analysis and Data Processing

Analyses of lipid samples were carried out using HPLC coupled with electrospray ionization tandem mass spectrometry (HPLC ESI-MS-MS), as described previously [64]. The Lipidyzer platform consisting of a Nexera X2 HPLC (Shimadzu Germany GmbH, Duisburg, Germany) and a Sciex QTRAP 5500 system equipped with SelexION technology (AB Sciex Germany GmbH, Darmstadt, Germany) was used for lipidomic analysis. The nomenclature of lipids proposed by the Lipid Maps Consortium was used in this study [67].

### 4.5. Statistical Analysis

The Shapiro–Wilk test was used to evaluate the normality of the data. Binary logistic regression modeling was used to analyze the association between levels of lipid species and the prognostic factors (presence/absence of metastasis, death, localization of metastasis) as dichotomous covariates with adjustment for age, sex, and type of therapy (none, immunotherapy, or targeted therapy). Odds ratios (ORs) and 95% confidence intervals (C.I.s) were calculated. Stepwise regression analysis with a forward selection was performed to identify lipid panels with a significant association with the presence of prognostic factors, adjusted for sex, age, and therapy approach. Receiver operating characteristic (ROC) curves were constructed from the logistic regression model, and the area under the curve (AUC) was used to assess the classification performance of the model. Using the AUC values, the Youden statistic was applied to find the cutoff point with the best sensitivity and specificity values [26]. For statistical calculation, the optimal cutoff point was considered to be the one with the highest value of the Youden index. The comparison of the lipid levels was calculated with the Mann–Whitney Wilcoxon test and Kruskal–Wallis tests followed by the Dunn’s test post hoc method.

Statistical analyses were carried out using IBM SPSS Statistics 26.0 software (IBM company, Palo Alto, CA, USA) or R 3.6.1 software (R Foundation for Statistical Computing, Vienna, Austria). *p* < 0.05 was considered statistically significant.

## Data Availability

Dataset available on request from the authors.

## References

[1] Ning B., Liu Y., Wang M., Li Y., Xu T., We Y. (2022). The Predictive Value of Tumor Mutation Burden on Clinical Efficacy of Immune Checkpoint Inhibitors in Melanoma: A Systematic Review and Meta-Analysis. Front. Pharmacol..

[2] Hanahan D., Weinberg R.A. (2011). Hallmarks of cancer: The next generation. Cell.

[3] Warburg O., Wind F., Negelein E. (1927). The Metabolism of Tumors in the Body. J. Gen. Physiol..

[4] Swinnen J.V., Brusselmans K., Verhoeven G. (2006). Increased lipogenesis in cancer cells: New players, novel targets. Curr. Opin. Clin. Nutr. Metab. Care.

[5] Spector A.A., Steinberg D. (1966). Relationship between fatty acid and glucose utilization in Ehrlich ascites tumor cells. J. Lipid Res..

[6] Tator C.H., Evans J.R., Olszewski J. (1966). Tracers for the detection of brain tumors. Evaluation of radioiodinated human serum albumin and radioiodinated fatty acid. Neurology.

[7] Jensen V., Ladekarl M., Holm-Nielsen P., Melsen F., Soerensen F.B. (1995). The prognostic value of oncogenic antigen 519 (OA-519) expression and proliferative activity detected by antibody MIB-1 in node-negative breast cancer. J. Pathol..

[8] Butler L.M., Perone Y., Dehairs J., Lupien L.E., de Laat V., Talebi A., Loda M., Kinlaw W.B., Swinnen J.V. (2020). Lipids and cancer: Emerging roles in pathogenesis, diagnosis and therapeutic intervention. Adv. Drug Deliv. Rev..

[9] Hao Y., Li D., Xu Y., Ouyang J., Wang Y., Zhang Y., Li B., Xie L., Qin G. (2019). Investigation of lipid metabolism dysregulation and the effects on immune microenvironments in pan-cancer using multiple omics data. BMC Bioinform..

[10] Peck B., Schulze A. (2019). Lipid Metabolism at the Nexus of Diet and Tumor Microenvironment. Trends Cancer.

[11] Nagarajan S.R., Butler L.M., Hoy A.J. (2021). The diversity and breadth of cancer cell fatty acid metabolism. Cancer Metab..

[12] Riscal R., Skuli N., Simon M.C. (2019). Even Cancer Cells Watch Their Cholesterol!. Mol. Cell.

[13] Mollinedo F., Gajate C. (2020). Lipid rafts as signaling hubs in cancer cell survival/death and invasion: Implications in tumor progression and therapy: Thematic Review Series: Biology of Lipid Rafts. J. Lipid Res..

[14] Kim H.Y., Lee H., Kim S.H., Jin H., Bae J., Choi H.K. (2017). Discovery of potential biomarkers in human melanoma cells with different metastatic potential by metabolic and lipidomic profiling. Sci. Rep..

[15] Guo Y., Wang X., Qiu L., Qin X., Liu H., Wang Y., Li F., Wang X., Chen G., Song G. (2012). Probing gender-specific lipid metabolites and diagnostic biomarkers for lung cancer using Fourier transform ion cyclotron resonance mass spectrometry. Clin. Chim. Acta.

[16] Patel N., Vogel R., Chandra-Kuntal K., Glasgow W., Kelavkar U. (2014). A novel three serum phospholipid panel differentiates normal individuals from those with prostate cancer. PLoS ONE.

[17] Cas M.D., Ciniselli C.M., Vergani E., Ciusani E., Aloisi M., Duroni V., Verderio P., Ghidoni R., Paroni R., Perego P. (2024). Alterations in Plasma Lipid Profiles Associated with Melanoma and Therapy Resistance. Int. J. Mol. Sci..

[18] Fu Y., Zou T., Shen X., Nelson P.J., Li J., Wu C., Yang J., Zheng Y., Bruns C., Zhao Y. (2021). Lipid metabolism in cancer progression and therapeutic strategies. MedComm.

[19] Aloia A., Müllhaupt D., Chabbert C.D., Eberhart T., Flückiger-Mangual S., Vukolic A., Eichhoff O., Irmisch A., Alexander L.T., Scibona E. (2019). A Fatty Acid Oxidation-dependent Metabolic Shift Regulates the Adaptation of BRAF-mutated Melanoma to MAPK Inhibitors. Clin. Cancer Res..

[20] Rysman E., Brusselmans K., Scheys K., Timmermans L., Derua R., Munck S., Van Veldhoven P.P., Waltregny D., Daniëls V.W., Machiels J. (2010). De novo lipogenesis protects cancer cells from free radicals and chemotherapeutics by promoting membrane lipid saturation. Cancer Res..

[21] Wang H., Franco F., Tsui Y.C., Xie X., Trefny M.P., Zappasodi R., Mohmood S.R., Fernández-García J., Tsai C.H., Schulze I. (2020). CD36-mediated metabolic adaptation supports regulatory T cell survival and function in tumors. Nat. Immunol..

[22] Stephenson D.J., Hoeferlin L.A., Chalfant C.E. (2017). Lipidomics in translational research and the clinical significance of lipid-based biomarkers. Transl. Res..

[23] Perrotti F., Rosa C., Cicalini I., Sacchetta P., Del Boccio P., Genovesi D., Pieragostino D. (2016). Advances in Lipidomics for Cancer Biomarkers Discovery. Int. J. Mol. Sci..

[24] Cvetkovic Z., Cvetkovic B., Petrovic M., Ranic M., Debeljak-Martarcic J., Vucic V., Glibetic M. (2009). Lipid profile as a prognostic factor in cancer patients. J BUON.

[25] Muntoni S., Atzori L., Mereu R., Satta G., Macis M.D., Congia M., Tedde A., Desogus A., Muntoni S. (2009). Serum lipoproteins and cancer. Nutr. Metab. Cardiovasc. Dis..

[26] Ghahremanfard F., Mirmohammadkhani M., Shahnazari B., Gholami G., Mehdizadeh J. (2015). The Valuable Role of Measuring Serum Lipid Profile in Cancer Progression. Oman Med. J..

[27] Li R., Liu B., Liu Y., Liu Y., He Y., Wang D., Sun Y., Xu Y., Yu Q. (2020). Elevated serum lipid level can serve as early signal for metastasis for Non-Small Cell Lung Cancer patients: A retrospective nested case-control study. J. Cancer.

[28] Zhang L., Han L., He J., Lv J., Pan R., Lv T. (2020). A high serum-free fatty acid level is associated with cancer. J. Cancer Res. Clin. Oncol..

[29] Fan L., Lin Q., Huang X., Fu D., Huang H. (2021). Prognostic significance of pretreatment serum free fatty acid in patients with diffuse large B-cell lymphoma in the rituximab era: A retrospective analysis. BMC Cancer.

[30] Zhang Y., Song L., Liu N., He C., Li Z. (2014). Decreased serum levels of free fatty acids are associated with breast cancer. Clin. Chim. Acta.

[31] Hannun Y.A., Obeid L.M. (2018). Sphingolipids and their metabolism in physiology and disease. Nat. Rev. Mol. Cell Biol..

[32] Faedo R.R., da Silva G., da Silva R.M., Ushida T.R., da Silva R.R., Lacchini R., Matos L.L., Kowalski L.P., Lopes N.P., Leopoldino A.M. (2022). Sphingolipids signature in plasma and tissue as diagnostic and prognostic tools in oral squamous cell carcinoma. Biochim. Biophys. Acta-Mol. Cell Biol. Lipids.

[33] Nath A., Li I., Roberts L.R., Chan C. (2015). Elevated free fatty acid uptake via CD36 promotes epithelial-mesenchymal transition in hepatocellular carcinoma. Sci. Rep..

[34] Nath A., Chan C. (2016). Genetic alterations in fatty acid transport and metabolism genes are associated with metastatic progression and poor prognosis of human cancers. Sci. Rep..

[35] Pandey V., Vijayakumar M.V., Ajay A.K., Malvi P., Bhat M.K. (2012). Diet-induced obesity increases melanoma progression: Involvement of Cav-1 and FASN. Int. J. Cancer.

[36] Ueno G., Iwagami Y., Kobayashi S., Mitsufuji S., Yamada D., Tomimaru Y., Akita H., Asaoka T., Noda T., Gotoh K. (2022). CAT-1-Regulated Cholesteryl Ester Accumulation Modulates Gemcitabine Resistance in Biliary Tract Cancer. Ann. Surg. Oncol..

[37] Chang T.Y., Chang C.C., Cheng D. (1997). Acyl-coenzyme A:cholesterol acyltransferase. Annu. Rev. Biochem..

[38] Phillips M.C. (2014). Molecular mechanisms of cellular cholesterol efflux. J. Biol. Chem..

[39] Hartmann P., Trufa D.I., Hohenberger K., Tausche P., Trump S., Mittler S., Geppert C.I., Rieker R.J., Schieweck O., Sirbu H. (2023). Contribution of serum lipids and cholesterol cellular metabolism in lung cancer development and progression. Sci. Rep..

[40] Jiang J., Nilsson-Ehle P., Xu N. (2006). Influence of liver cancer on lipid and lipoprotein metabolism. Lipids Health Dis..

[41] Motta M., Giugno I., Ruello P., Pistone G., Di Fazio I., Malaguarnera M. (2001). Lipoprotein (a) behaviour in patients with hepatocellular carcinoma. Minerva Med..

[42] Argiles J.M., Lopez-Soriano F.J., Evans R.D., Williamson D.H. (1989). Interleukin-1 and lipid metabolism in the rat. Biochem. J..

[43] Kwong L.K., Ridinger D.N., Bandhauer M., Ward J.H., Samlowski W.E., Iverius P.H., Pritchard H., Wilson D.E. (1997). Acute dyslipoproteinemia induced by interleukin-2: Lecithin: Cholesteryl acyltransferase, lipoprotein lipase, and hepatic lipase deficiencies. J. Clin. Endocrinol. Metab..

[44] Zimmerman W.F., Keys S. (1989). Lysophospholipase and the metabolism of lysophosphatidylcholine in isolated bovine rod outer segments. Exp. Eye Res..

[45] Liu P., Zhu W., Chen C., Yan B., Zhu L., Chen X., Peng C. (2020). The mechanisms of lysophosphatidylcholine in the development of diseases. Life Sci..

[46] Kuliszkiewicz-Janus M., Tuz M.A., Baczynski S. (2005). Application of 31P MRS to the analysis of phospholipid changes in plasma of patients with acute leukemia. Biochim. Biophys. Acta.

[47] Murphy R.A., Wilke M.S., Perrine M., Pawlowicz M., Mourtzakis M., Lieffers J.R., Maneshgar M., Bruera E., Clandinin M.T., Baracos V.E. (2010). Loss of adipose tissue and plasma phospholipids: Relationship to survival in advanced cancer patients. Clin. Nutr..

[48] Süllentrop F., Moka D., Neubauer S., Haupt G., Engelmann U., Hahn J., Schicha H. (2002). 31P NMR spectroscopy of blood plasma: Determination and quantification of phospholipid classes in patients with renal cell carcinoma. NMR Biomed..

[49] Murphy R.A., Bureyko T.F., Mourtzakis M., Chu Q.S., Clandinin M.T., Reiman T., Mazurak V.C. (2012). Aberrations in plasma phospholipid fatty acids in lung cancer patients. Lipids.

[50] Zhao Z., Xiao Y., Elson P., Tan H., Plummer S.J., Berk M., Aung P.P., Lavery I.C., Achkar J.P., Li L. (2007). Plasma lysophosphatidylcholine levels: Potential biomarkers for colorectal cancer. J. Clin. Oncol..

[51] Ojala P.J., Hirvonen T.E., Hermansson M., Somerharju P., Parkkinen J. (2007). Acyl chain-dependent effect of lysophosphatidyl-choline on human neutrophils. J. Leukoc. Biol..

[52] Brkic L., Riederer M., Graier W.F., Malli R., Frank S. (2012). Acyl chain-dependent effect of lysophosphatidylcholine on cyclooxygenase (COX)-2 expression in endothelial cells. Atherosclerosis.

[53] Kang S., Kim M.H., Park I.A., Kim J.W., Park N.H., Kang D., Yoo K.Y., Kang S.B., Lee H.P., Song Y.S. (2006). Elevation of cyclooxygenase-2 is related to lymph node metastasis in adenocarcinoma of uterine cervix. Cancer Lett..

[54] Kyzas P.A., Stefanou D., Agnantis N.J. (2005). COX-2 expression correlates with VEGF-C and lymph node metastases in patients with head and neck squamous cell carcinoma. Mod. Pathol..

[55] Crofford L.J. (1997). COX-1 and COX-2 tissue expression: Implications and predictions. J. Rheumatology. Suppl..

[56] Denkert C., Köbel M., Berger S., Siegert A., Leclere A., Trefzer U., Hauptmann S. (2001). Expression of cyclooxygenase 2 in human malignant melanoma. Cancer Res..

[57] Zhou P., Qin J., Li Y., Li G., Wang Y., Zhang N., Chen P., Li C. (2017). Combination therapy of PKCzeta and COX-2 inhibitors synergistically suppress melanoma metastasis. J. Exp. Clin. Cancer Res..

[58] Tudor D.V., Bâldea I., Lupu M., Kacso T., Kutasi E., Hopârtean A., Stretea R., Gabriela Filip A. (2020). COX-2 as a potential biomarker and therapeutic target in melanoma. Cancer Biol. Med..

[59] Gerl M.J., Vaz W.L.C., Domingues N., Klose C., Surma M.A., Sampaio J.L., Almeida M.S., Rodrigues G., Araújo-Gonçalves P., Ferreira J. (2018). Cholesterol is Inefficiently Converted to Cholesteryl Esters in the Blood of Cardiovascular Disease Patients. Sci. Rep..

[60] Liu Z., Gomez C.R., Espinoza I., Le T.P.T., Shenoy V., Zhou X. (2022). Correlation of cholesteryl ester metabolism to pathogenesis, progression and disparities in colorectal Cancer. Lipids Health Dis..

[61] Tisdale M.J. (2002). Cachexia in cancer patients. Nat. Rev. Cancer.

[62] Zwickl H., Hackner K., Köfeler H., Krzizek E.C., Muqaku B., Pils D., Scharnagl H., Solheim T.S., Zwickl-Traxler E., Pecherstorfer M. (2020). Reduced LDL-Cholesterol and Reduced Total Cholesterol as Potential Indicators of Early Cancer in Male Treatment-Naive Cancer Patients With Pre-cachexia and Cachexia. Front. Oncol..

[63] Zhang X., Zhao X.W., Liu D.B., Han C.Z., Du L.L., Jing J.X., Wang Y. (2014). Lipid levels in serum and cancerous tissues of colorectal cancer patients. World J. Gastroenterol..

[64] Pikó P., Pál L., Szűcs S., Kósa Z., Sándor J., Ádány R. (2021). Obesity-Related Changes in Human Plasma Lipidome Determined by the Lipidyzer Platform. Biomolecules.

[65] Franko A., Merkel D., Kovarova M., Hoene M., Jaghutriz B.A., Heni M., Königsrainer A., Papan C., Lehr S., Häring H.U. (2018). Dissociation of Fatty Liver and Insulin Resistance in I148M PNPLA3 Carriers: Differences in Diacylglycerol (DAG) FA18:1 Lipid Species as a Possible Explanation. Nutrients.

[66] Ubhi B.K. (2018). Direct Infusion-Tandem Mass Spectrometry (DI-MS/MS) Analysis of Complex Lipids in Human Plasma and Serum Using the Lipidyzer Platform. Clin. Metabolomics Methods Protoc..

[67] Liebisch G., Fahy E., Aoki J., Dennis E.A., Durand T., Ejsing C.S., Fedorova M., Feussner I., Griffiths W.J., Köfeler H. (2020). Update on LIPID MAPS classification, nomenclature, and shorthand notation for MS-derived lipid structures. J. Lipid Res..

